# Quantification of Aortic Valve Calcification in Contrast-Enhanced Computed Tomography

**DOI:** 10.3390/jcm13082386

**Published:** 2024-04-19

**Authors:** Danai Laohachewin, Philipp Ruile, Philipp Breitbart, Jan Minners, Nikolaus Jander, Martin Soschynski, Christopher L. Schlett, Franz-Josef Neumann, Dirk Westermann, Manuel Hein

**Affiliations:** 1 Department of Cardiology and Angiology, Medical Center—University of Freiburg, Faculty of Medicine, Suedring 15, 79189 Bad Krozingen, Germany; 2 Department of Diagnostic and Interventional Radiology, Medical Center—University of Freiburg, Faculty of Medicine, University of Freiburg, Hugstetter Straße 55, 79106 Freiburg, Germany

**Keywords:** aortic valve stenosis, contrast-enhanced computed tomography, aortic valve calcification

## Abstract

**Background**: The goal of our study is to evaluate a method to quantify aortic valve calcification (AVC) in contrast-enhanced computed tomography for patients with suspected severe aortic stenosis pre-interventionally. **Methods**: A total of sixty-five patients with aortic stenosis underwent both a native and a contrast-enhanced computed tomography (CECT) scan of the aortic valve (45 in the training cohort and 20 in the validation cohort) using a standardized protocol. Aortic valve calcification was semi-automatically quantified via the Agatston score method for the native scans and was used as a reference. For contrast-enhanced computed tomography, a calcium threshold of the Hounsfield units of the aorta plus four times the standard deviation was used. **Results**: For the quantification of aortic valve calcification in contrast-enhanced computed tomography, a conversion formula (691 + 1.83 x AVCCECT) was derived via a linear regression model in the training cohort. The validation in the second cohort showed high agreement for this conversion formula with no significant proportional bias (Bland–Altman, *p* = 0.055) and with an intraclass correlation coefficient in the validation cohort of 0.915 (confidence interval 95% 0.786–0.966) *p* < 0.001. **Conclusions**: Calcium scoring in patients with aortic valve stenosis can be performed using contrast-enhanced computed tomography with high validity. Using a conversion factor led to an excellent agreement, thereby obviating an additional native computed tomography scan. This might contribute to a decrease in radiation exposure.

## 1. Introduction

Aortic valve stenosis is the most common degenerative valve pathology in the western world, characterized by progressive valve thickening, calcification and stiffening, leading to orifice obstruction [1,2]. The measurement of aortic valve calcification has been demonstrated to be an increasingly powerful tool to predict adverse events. It has been shown that the moderate-to-severe calcification of the aortic valve is a strong independent risk factor for unfavorable cardiovascular outcomes even in asymptomatic patients [3,4]. Furthermore, its preinterventional assessment serves as a prognostic factor for postinterventional events such as pacemaker implantation due to relevant conduction disturbances and paravalvular regurgitation after transcatheter aortic valve implantation (TAVI) [5,6,7]. Thus, the value of aortic valve calcium measurement is valuable for several reasons.

Suspected severe symptomatic aortic stenosis requires further diagnostics for treatment planning. The current echocardiographic definition based on the 2021 guidelines from the European Society of Cardiology for severe aortic valve stenosis requires the measurement of the mean gradient to be equal or greater than 40 mmHg, a peak velocity of greater than 4 m/s and the estimated valve area of the aortic valve through a continuity formula of less than 1 cm^2^. Low gradient (mean gradient <40 mmHg) aortic valve stenosis is sometimes difficult to quantify especially in patients with normal ejection fraction (>50%) and low stroke volumes (<35 mL/m^2^). Therefore, in borderline cases, additional imaging is recommended to estimate the actual severity of aortic valve stenosis [8].

If a low-flow low-gradient aortic valve stenosis is suspected, current European guidelines recommend the multimodal imaging acquisition of supporting evidence of severe stenosis such as stress echocardiography (in left ventricular ejection fraction <50%) and computed tomography measurements of aortic valve calcification [8,9]. Dobutamine stress echocardiography is recommended to discriminate between severe and pseudosevere low-flow stenosis, if ejection fraction is reduced. However, if there is no flow reserve under dobutamine or there is a normal flow (>35 mL/m^2^), the quantification of aortic valve calcification via computed tomography is the recommended next step [2,8]. Initially proposed by Agatston et al. for coronary artery calcium quantification, this method can be applied to the aortic valve by acquiring non-contrast scans at 120 kilovolts of the native valve [10,11,12]. The anatomic correlation and validation of calcium content compared to in vivo computed tomography scoring was excellent, thus confirming its reliability to accurately measure aortic valve calcification [13].

There are scarce amounts of experiences in the quantification of aortic valve calcification in contrast-enhanced images. As there is a growing interest to reduce the necessary pre-interventional workup and radiation dose, there are attempts to quantify the calcification load in contrast-enhanced images, which are necessary for pre-interventional planning prior to transcatheter aortic valve implantation and to omit native scans [1]. Initial correction formulas in contrast-enhanced computed tomography have been proposed to estimate calcification burden. To potentially reduce scan time and radiation exposure, they proposed the use of contrast-enhanced imaging with a dynamic cut-off for calcium detection. They used the average attenuation of a distinct area in the ascending aorta and added two-to-three times the standard deviation of attenuation in this area for calcium detection. Using contrast-enhanced TAVI-planning images has the advantage of a higher resolution and finer layer thickness [14,15].

Therefore, to reduce radiation exposure and increase accuracy, we sought to establish a conversion factor to calculate the Agatston score of aortic valves by using contrast-enhanced images with higher thresholds for overall noise reduction.

## 2. Materials and Methods

### 2.1. Patient Population

For this retrospective study, a total of 65 patients with available pre-TAVI-computed tomographic data were screened and recruited between June and July of 2021. Patients with a previous aortic valve replacement, aortic dissections or aneurysms and pre-procedural pacemaker implantations were excluded from the study. The cohort was split into a derivation (*n* = 45) and a validation cohort (*n* = 20) by random selection. Baseline characteristics include clinical and echocardiographic data as well as the radiation and contrast agent quantities from the computed tomography examination. Local institutional review board approval compliant with the Declaration of Helsinki was obtained.

### 2.2. Computed Tomography Acquisition and Reconstruction

All patients underwent non-contrast as well as contrast-enhanced computed tomography on a second-generation dual-source scanner (Somatom Definition Flash, Siemens Healthcare, Forchheim, Germany). For contrast-enhanced imaging, an iodinated contrast agent was used (Imeron 400, Bracco, Konstanz, Germany). All images were reconstructed at 50 ms steps throughout the cardiac cycle with a slice thickness of 1 mm and an increment of 0.75 mm using a medium soft tissue convolution kernel (B26f). Image processing and analysis were performed on a dedicated post-processing workstation (Syngo Multimodality Workplace, Siemens Healthcare, Forchheim, Germany) with quantification using 3mensio Workstation Version 10.1 (Pie Medical Imaging BV, Maastricht, The Netherlands). A detailed protocol for ECG-gated cardiac computed tomography angiography was described previously [16].

### 2.3. Agatston Score

**Figure 1:**
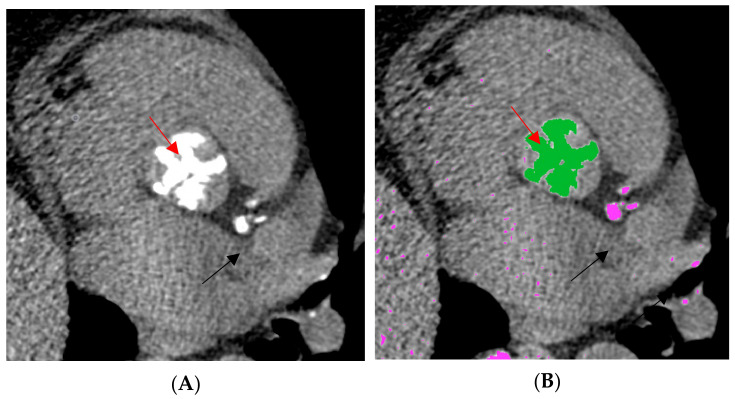
Sample images of native computed tomography showing aortic calcification with the semi-automatic detection of aortic valve calcification with an attenuation threshold of ≥130 Hounsfield units. (**A**): A two-dimensional representation of the aortic valve in non-contrast computed tomography. (**B**): A semi-automatic quantification for the Agatston calculation; green = the semi-automatic selection of the aortic valve; pink = detections of calcification at a predetermined threshold of ≥130 Hounsfield units; red arrow = aortic valve; black arrow = calcified structures such as coronary arteries.

Quantification of aortic valve calcification was done by applying the Agatston score on native scans with a 3 mm slice thickness. Predefined settings with ≥130 Hounsfield units at 120 kilovolts were used for the selection of defined areas of calcification [1,10]. The exclusion of other adjacent calcified structures such as coronary arteries or proximal aorta was done manually after careful selection (Figure 1).

### 2.4. Threshold Measurement in Contrast-Enhanced Computed Tomography

**Figure 2:**
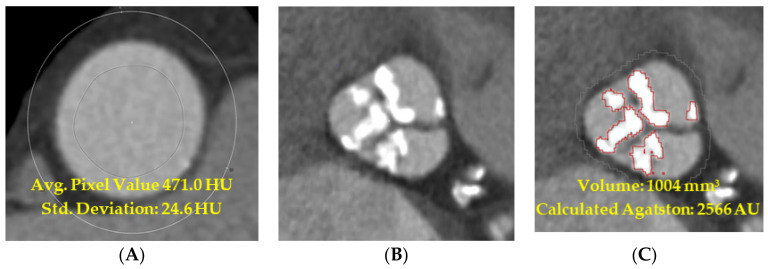
Sample images of a contrast-enhanced computed tomography showing aortic calcification. (**A**) The dynamic threshold is determined by measuring the attenuation of the ascending aorta adding four times the standard deviation. (**B**) A visualization of calcification using the new threshold. (**C**) This new threshold is then used for the semi-automatic detection of aortic calcification. Red-bordered structures = semi-automatic quantifications of a calcified aortic valve.

For the threshold determination of calcification on contrast-enhanced images, the contrast attenuation in Hounsfield units was measured by drawing a circular region of interest (ROI) covering the central 2/3 of the area of the vessel lumen at 3 cm above the annulus of the aortic valve in the ascending aorta. The average attenuation of the ROI with the addition of four times the standard deviation of each ROI was used as a new threshold for the segmentation of aortic valve calcification in contrast-enhanced imaging. This led to the calcification volumes of the contrast-enhanced images (Figure 2).

### 2.5. Predictive Quantification of Aortic Valve Calcification in Contrast-Enhanced Computed Tomography

For non-contrast computed tomography, the Agatston method was used for the quantification of aortic valve calcification as described above [4]. For contrast-enhanced computed tomography, the individual threshold for calcification was applied. The resulting semi-automatic segmentation of the aortic valve calcification was quantified after the correction for adjacent structures described above.

### 2.6. Statistical Analysis

Continuous variables are presented as means with standard deviation. Categorical variables are presented as counts and percentages. The derivation cohort was used to generate a linear regression model of the Agatston score derived from native scans and the calcification volume derived from contrast-enhanced scans. The formula of the linear regression analysis was subsequently tested in the validation cohort. The reliability of this conversion formula was evaluated by an inter-class correlation coefficient and the construction of Bland–Altman plots to assess the agreement between the two measuring methods [17]. Statistical significance was defined as *p* < 0.05. All analyses were performed on statistical software package SPSS, Version 23 (IBM Corp., Armonk, NY, USA).

## 3. Results

### 3.1. Patient Demographics

A total of 65 subjects were included in the study. Forty-five were randomly assigned into the derivation cohort and twenty into the validation cohort. The derivation and validation cohort contained 22 and 6 female patients, respectively. The mean age in years in the derivation and validation cohort was 81.0 ± 6.2 and 78.5 ± 5.0, respectively. In the derivation cohort, the Agatston score of native computed tomography had a median of 2714.5 with an interquartile range of 1840.8–3917.3, and the calculated Agatston of contrast-enhanced computed tomography had a median of 2738.7 with an interquartile range of 1814.6–3636.4, respectively. In the validation cohort, the Agatston score of native computed tomography had a median of 2123.0 with an interquartile range of 1386.0–3072.0 and the calculated Agatston of contrast-enhanced computed tomography had a median of 2137.6 with an interquartile range of 1588.2–2543.4, respectively. The demographical data and the characteristics of the computed tomographic scans are listed in Table 1.

### 3.2. Derivation of the Conversion Formula to Calculate the Agatston Score in Contrast-Enhanced Computed Tomography Scans

**Figure 3:**
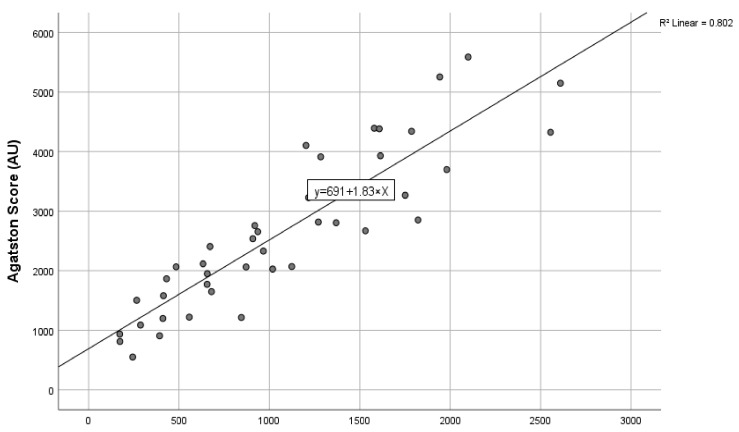
The correlation of aortic valve calcification quantification on contrast-enhanced computed tomography and the standard Agatston score on non-contrast-enhanced computed tomography with the line of best fit.

Our linear regression model using the line of best fit yielded an Agatston score conversion formula of calculated Agatston units = 691 + 1.83 × AVC (contrast-enhanced computed tomography) mm^3^. The agreement of the interclass correlation coefficient of this cohort was 0.942 (confidence interval 95% 0.894–0.968) *p* < 0.001. The linear regression analysis is shown in Figure 3.

### 3.3. Confirmation of Agreement in the Validation Cohort

**Figure 4:**
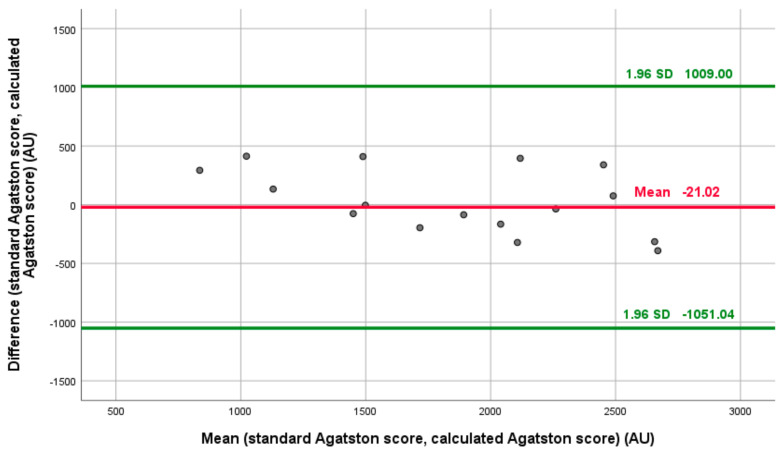
The Bland–Altman plot comparing the standard Agatston score versus the calculated contrast-enhanced imaging-derived Agatston score for aortic valve calcium quantification (*p* = 0.055).

Next, the formula was validated. The derived conversion formula achieved a high agreement with an interclass coefficient of 0.915 (confidence interval 95% 0.786–0.966), *p* < 0.001. The Bland–Altman analysis comparing both standard and predicted Agatston scores in the validation cohort revealed no proportional bias (*p* = 0.055). The Bland–Altman and correlation analyses are shown in Figure 4 and Table 2.

## 4. Discussion

### 4.1. Main Findings

We generated a simple conversion formula as a practicable and accurate approach to quantify aortic valve calcification in contrast-enhanced computed tomography. In our study, we employed a higher cut-off for calcium detection of plus four times the standard deviation. In comparison to the approaches of Alqahtani et al. and Eberhardt et al. [14,15], the technical advantages of our approach are its higher spatial resolution with a better differentiation of paravalvular structures such as annular or aortic vessel calcification or ostial coronary stents [16]. We derived a linear correlation formula with excellent correlation to native Agatston scores and a high validity verified by Bland–Altman calculations as described above.

### 4.2. Comparison with Previous Data

The quantification of aortic valve calcification is routinely performed on non-contrast-enhanced computed tomography scans. The results have potential diagnostic and prognostic value for patients with aortic valve stenosis, especially in patients with a reduced echocardiographic window or low-flow/low-gradient stenosis [8,18]. As the number of aortic valve interventions is growing, so will the number and, thus, the cost of pre-interventional diagnostics. Previous studies have shown that it is possible to estimate the Agatston score by using contrast-enhanced CT scans. Those studies presented similar results by using slightly different approaches and conversion formulas. Alqahtani et al. derived a linear correlation by defining calcified volumes two times above the standard deviation of the contrast enhanced aortic vessel with a function forced to cross zero. Their population had varying degrees of aortic stenosis [15]. Eberhard et al. presented a pre-interventional population, thus having a cohort with a higher degree of aortic stenosis with a more complex conversion factor resulting in a slightly convex correlation. They applied three times the standard deviation for their definition of calcified volumes. Even by applying a simpler equation to achieve the Agatston score by multiplying the calcification volume of contrast-enhanced images by two, there was a minor reduction of agreement compared to their formula [14]. 

Our results have revealed similarly high agreements with an approach that is less susceptible to artifacts by using four times the standard deviation for the definition of the calcified volume by firstly being linear and secondly being about the range of an upward slope of two as seen in previous studies. Similar to Eberhard et al., as we specifically recruited pre-interventional patients with higher degrees of aortic stenosis, our line of best fit does not cross at zero, as we have no data on patients with no or mildly aortic stenosis. Thus, even though the approaches to determining aortic valve calcifications were different, our method has shown excellent validity and reliability similar to those of Alqahtani et al. and Eberhard et al. [14,15].

An alternative method for the quantification of calcification volumes was presented by Abdelkhalek et al. Their method automatically detects a threshold to minimize the false positive rate [19,20]. This method provides an attenuation stable calcification detection threshold and is supposed to be independent of the luminal fluctuations of contrast information. Though unique, a correlation or conversion to the classical Agatston score for prognostic patient evaluation as well as an external validation are lacking so far [19,20].

### 4.3. Importance of Agatston Score as a Prognostic Tool

As described before, quantification of aortic valve calcification using the Agatston method has been shown to be a valuable tool as a risk factor for several events. The presence of aortic valve calcification alone is associated with a significantly higher risk for cardiovascular and coronary events [1,4]. A combination of elevated aortic valve calcium content and a pre-existing complete right bundle branch block has led to a higher risk for pacemaker implantation according to several authors [5,21,22,23]. Total aortic valve calcification has been implicated for a higher risk of paravalvular leakage after intervention [24,25,26,27]. There is currently no data on the calculated Agatston acquired through contrast-enhanced computed tomography and its impact on clinical outcomes. Only Kong et al. has shown a positive association of paravalvular leakage after intervention using a logarithmic transformed Agatston score [27]. As our conversion factor has shown a linear trend, similarly to Alqahtani et al. and Eberhard et al. in their rule of thumb of two times the aortic valve calcification segmented on contrast-enhanced computed tomography, one can postulate that the calculated Agatston score would predict clinical outcomes similarly to the native Agatston score [14,15]. Further validation would be required to confirm that hypothesis. 

### 4.4. Pixel Noise Detection

Pixel noise detection is a limiting factor in accurately identifying calcified lesions in contrast-enhanced computed tomography even with advances in protocols and techniques for improved efficacy [28,29]. A classic Agatston score is derived from 3 mm slices, thus reducing the precision of measurement of aortic valve calcification and potentially generating more noise from the surrounding structures [10]. We have chosen to add four times the standard deviation for visual improvement in contrast-enhanced imaging of items such as large vessels. Furthermore, the use of a 0.75 mm slice thickness results in a better delineation of calcified non-valvular structures even in semi-automatic quantification of the calcification of the aortic valve and its surrounding structures per our experience. In line with the mentioned publications, this led to a high level of agreement in our calculations. Further prospective studies with larger cohorts should be discussed to explore a potential prognostic use of this method. 

### 4.5. Fixed and Dynamic Thresholds for the Quantification of Aortic Valve Calcification

The standardized threshold of 130 HU for calcification in non-contrast-enhanced computed tomography remains universal since the introduction of the Agatston method in 1990, even though it was originally developed for coronary calcification measurement [10]. Limited data are available for calcium threshold determination in contrast-enhanced imaging. Fixed and dynamic thresholds of the specified Hounsfield units for calcium detection in contrast-enhanced computed imaging have been suggested. The degree of opacification in contrast-enhanced computed tomography is dependent on multiple factors such as patient size and cardiac output, scanner settings and contrast medium use. For fixed values, several studies have used a range of 300–850 Hounsfield units [30]. For dynamic thresholds, values are acquired using the luminal attenuation in the ascending aorta adding two-to-four times the standard deviation [2]. There is currently no standardized protocol to acquire and measure calcification in contrast-enhanced computed tomography. As with the previous investigators, we have shown the high agreement of our conversion formula using a dynamic threshold. Whether fixed intensity values yield the same accuracy as dynamic intensity values has not been investigated yet.

### 4.6. Study Limitations

Some limitations should be noted. Firstly, our data included only patients who were investigated specifically for suspected higher-grade aortic valve stenosis. Thus, the conversion formula was not designed on low to possibly mid-grade aortic valve stenosis. Therefore, it can be applied with high accuracy only within this specific group. Secondly, even with high agreement in the internal validation cohort, the conversion formula was based on a small sample population. Lastly, the conformity of this data may not apply to different centers and regions due to different computed tomography scanners, image acquisition protocols and contrast medium usages. Supplementarily, we did not acquire prognostic data, making such data an intriguing area for further exploration. 

## 5. Conclusions

Our study has shown that the approximation of the Agatston score of the aortic valve using a conversion factor in contrast-enhanced computed tomography is a useful alternative. It provides excellent image quality and nullifies the need for additional native imaging, thus reducing patients’ exposure to radiation. Further clinical evidence in terms of studies with larger cohorts and prognostic data should follow to support this method.

## Figures and Tables

**Table 1:** Patient demographics.

Demographics	Derivation Cohort (*n* = 45)	Validation Cohort (*n* = 20)	*p*
Age (years)	81.0 ± 6.2	78.5 ± 5.0	0.472
Female (*n* (%))	22 (48.9%)	6 (30%)	0.156
Weight (kg)	75.2 ± 13.7	84.3 ± 16.6	0.023
Height (cm)	166.8 ± 7.9	166.6 ± 15.1	0.144
BMI (kg/m^2^)	27.0 ± 4.5	29.1 ± 4.9	0.094
Hypertension (*n* (%))	40 (89.0%)	15 (75.0%)	0.152
Diabetes (*n* (%))	15 (33.3%)	9 (45.0%)	0.368
Dyslipidaemia (*n* (%))	27 (60.0%)	15 (75.0%)	0.243
Smoking (*n* (%))	6 (13.3%)	5 (25.0%)	0.247
Coronary artery disease (*n* (%))	30 (66.7%)	12 (60.0%)	0.604
Renal disease (*n* (%))	7 (15.6%)	5 (25.0%)	0.365
Peripheral artery disease (*n* (%))	9 (20.0%)	5 (25.0%)	0.651
Non-contrast CT			
Tube voltage (kV)	120	120	
Tube current (mAs)	142.9 ± 29.4	152.8 ± 34.5	0.249
Contrast-enhanced CT			
Tube voltage (kV)	100 ± 0	100 ± 0	
Tube current (mAs)	480.7 ± 113.3	512.3 ± 111.5	0.320
AVC segmentation threshold (HU)	471 ± 117	461 ± 88	0.537

**Table 2:** Coefficient and correlation calculations.

Interclass correlation coefficient of the Derivation cohort	0.942 CI (0.894–0.968), *p* < 0.001
Interclass correlation coefficient of the Validation cohort	0.915 CI (0.786–0.966), *p* < 0.001
Goodness of fit (R^2^)	0.802, *p* < 0.001
Bland–Altman linear proportional bias P	0.055

## Data Availability

Transferring data to third parties is not included in the ethics statement. Please contact the corresponding author for submitting a request to the ethics committee, if desired.
